# Exclusively heteronuclear NMR experiments for the investigation of intrinsically disordered proteins: focusing on proline residues

**DOI:** 10.5194/mr-2-511-2021

**Published:** 2021-07-01

**Authors:** Isabella C. Felli, Wolfgang Bermel, Roberta Pierattelli

**Affiliations:** 1 CERM and Department of Chemistry “Ugo Schiff”, University of Florence, Via Luigi Sacconi 6, 50019 Sesto Fiorentino, Florence, Italy; 2 Bruker BioSpin GmbH, Silberstreifen 4, 76287 Rheinstetten, Germany

## Abstract

NMR represents a key spectroscopic technique that contributes to the emerging field of highly flexible, intrinsically disordered proteins (IDPs) or protein regions (IDRs) that lack a stable three-dimensional structure. A set of exclusively heteronuclear NMR experiments tailored for proline residues, highly abundant in IDPs/IDRs, are presented here. They provide a valuable complement to the widely used approach based on amide proton detection, filling the gap introduced by the lack of amide protons in proline residues within polypeptide chains. The novel experiments have very interesting properties for the investigations of IDPs/IDRs of increasing complexity.

## 1 Introduction

Invisible in X-ray studies of protein crystals, intrinsically disordered regions (IDRs) of complex proteins have for a long time been considered to be passive linkers connecting functional globular domains and are, thus, often ignored in structural biology studies. However, in many cases they comprise a significant fraction of the primary sequence of a protein, and for this reason, they are expected to have a role in protein function (Van Der Lee et al., 2014). The characterization of highly flexible regions of large proteins and entire proteins characterized by the lack of a 3D structure, now generally referred to as intrinsically disordered proteins (IDPs), lies well behind that of their folded counterparts and is nowadays pursued by an increasingly large number of studies to fill this knowledge gap. NMR plays a strategic role in this context since it constitutes the major, if not the unique, spectroscopic technique to achieve atomic resolution information on their structural and dynamic properties. However, intrinsic disorder and high flexibility have very relevant effects for NMR investigations, such as reduction in chemical shift dispersion and efficient exchange processes with the solvent due to the open conformations that, when approaching physiological pH and temperature, broaden amide proton resonances beyond detection. While several elegant experiments were proposed to exploit exchange processes with the solvent (Kurzbach et al., 2017; Olsen et al., 2020; Szekely et al., 2018; Thakur et al., 2013), in general initial NMR investigations of IDPs/IDRs are carried out in conditions in which these critical points are mitigated. Exchange broadening strongly depends on pH and temperature; conditions can be optimized to recover most of the amide proton resonances enabling the acquisition of amide-proton-detected triple resonance experiments needed for sequence-specific assignment of the resonances. However, in particular for proteins that are largely exposed to the solvent, it may be interesting to study their near-physiological pH and temperature conditions (Gil et al., 2013). In this context, ${}^{13}C$ direct detection NMR developed into a valuable alternative.

Although the intrinsic sensitivity of ${}^{13}C$ is lower with respect to that of ${}^{1}H$ , ${}^{13}C$ nuclear spins are characterized by a large chemical shift dispersion (Dyson and Wright, 2001) and, when coupled to ${}^{15}N$ nuclei, provide a well-defined fingerprint of a polypeptide (Bermel et al., 2006a; Hsu et al., 2009; Lopez et al., 2016; Schiavina et al., 2019). These features were exploited to design a suite of 3D experiments based on carbonyl-carbon direct detection for sequential assignment and to measure NMR observables (Felli and Pierattelli, 2014). These experiments, starting from ${}^{1}H$ polarization, exploit only heteronuclear chemical shifts in the indirect dimensions to maximize chemical shift dispersion (exclusively heteronuclear experiments) and can be used to study IDPs/IDRs – also in conditions in which amide proton resonances are too broad to be detected. In addition, they reveal information about proline residues that lack the amide proton when part of the polypeptide chains and cannot be detected in 2D ${}^{1}H{-}^{15}N$ correlation experiments (2D HN), even if pH and temperature conditions are optimized to reduce exchange broadening.

Proline residues are abundant in IDPs/IDRs and often occur in proline-rich sequences with repetitive units (Theillet et al., 2014). Initial bioinformatics studies on the relative abundance of each amino acid in regions of the protein that could not be observed in X-ray diffraction studies led to the classification of prolines as “disorder promoting” amino acids (Dunker et al., 2008). Nevertheless, proline, the only imino acid, features a closed ring in its side chain, which confers local rigidity compared to all other amino acids (Williamson, 1994), as also exploited in Förster Resonance Energy Transfer (FRET) studies in which proline residues are used as rigid spacers to measure distances (Schuler et al., 2005). These observations clearly show the importance of experimental atomic resolution information on the structural and dynamic properties of proline residues for understanding their role in modulating protein function. Abundant information about proline residues in globular protein folds is available either through NMR or X-ray studies (MacArthur and Thornton, 1991), including several examples of *cis*-trans isomerization of peptide bonds involving proline nitrogen as molecular switches (Lu et al., 2007). However, the characterization in highly flexible, disordered polypeptides is available only in a handful of cases (Chaves-Arquero et al., 2018; Gibbs et al., 2017; Haba et al., 2013; Hošek et al., 2016; Knoblich et al., 2009; Pérez et al., 2009; Piai et al., 2016; Chhabra et al., 2018; Ahuja et al., 2016), and actually, early studies on IDPs/IDRs routinely reported assignment statistics that only considered all other amino acids (“excluding prolines”).

Here we would like to propose an experimental variant of the most widely used ${}^{13}C$ -detected 3D experiments for the sequence-specific assignment of IDPs/IDRs to selectively pick up correlations involving proline nitrogen nuclei and provide key complementary information to that obtained through amide-proton-detected experiments. They can be collected in a shorter time with respect to standard 3D experiments and provide a valuable addition to the current experimental protocols for the study of IDPs.

## 2 Materials and methods

Isotopically labelled α -synuclein ( ${}^{13}C$ and ${}^{15}N$ ) was expressed and purified, as previously described (Huang et al., 2005). The NMR sample has 0.6 mM protein concentration in a 20 mM phosphate buffer at pH 6.5 and 100 mM NaCl in $H_{2}O$ with 5 % $D_{2}O$ for the lock signal.

Isotopically labelled CBP-ID4 ( ${}^{13}C$ and ${}^{15}N$ ) was expressed and purified as previously described (Piai et al., 2016). The NMR sample has 0.9 mM protein concentration in a water buffer containing 20 mM tris(hydroxymethyl)aminomethane (TRIS) and 50 mM KCl, at pH 6.9, with 5 % $D_{2}O$ added for the lock signal.

NMR experiments were acquired at 288 K (for α -synuclein) and at 283 K (for CBP-ID4) with a 16.4 T Bruker AVANCE NEO spectrometer operating at 700.06 MHz ${}^{1}H$ , 176.05 MHz ${}^{13}C$ , and 70.97 MHz ${}^{15}N$ frequencies, equipped with a 5 mm cryogenically cooled probe head optimized for ${}^{13}C$ direct detection (TXO). Radio frequency (RF) pulses and carrier frequencies typically employed for the investigation of intrinsically disordered proteins were used, except for the modifications introduced to zoom into the proline ${}^{15}N$ region. Carrier frequencies were set to 4.7 ppm (parts per million; ${}^{1}H$ ), 176.4 ( ${}^{13}C^{\prime }$ ), 53.9 ( ${}^{13}C^{\alpha }$ ), and 44.9 ( ${}^{13}C^{\mathrm{ali}}$ ). The ${}^{15}N$ carrier was set to 137 ppm in the centre of ${}^{15}N$ resonances of proline residues. Hard pulses were used for ${}^{1}H$ . Band-selective ${}^{13}C$ pulses used were Q5 and Q3 (Emsley and Bodenhausen, 1990) of 300 and 231 µs for 90 and 180 ${}^{\circ }$ rotations, respectively; a 900 µs Q3 pulse centred at 53.9 ppm was used for the selective inversion of $C^{\alpha }$ . The ${}^{15}N$ pulse to invert the ${}^{15}N$ proline resonances was a 8000 µs refocusing band-selective uniform-response pure-phase (RE-​BURP) pulse (Geen and Freeman, 1991); all other ${}^{15}N$ pulses were hard pulses. Decoupling was achieved with WALTZ-65 (100 µs , 2.5 kHz; Zhou et al., 2007) for ${}^{1}H$ and with GARP4 (250 µs , 1.0 kHz; Shaka et al., 1985) for ${}^{15}N$ . The MOCCA mixing time (Felli et al., 2009; Furrer et al., 2004) in the $(\mathrm{HCA}){\mathrm{COCON}}^{\mathrm{Pro}}$ experiment was 350 ms, constituted by repeated ( Δ -180 ${}^{\circ }$ - Δ ) ${}_{2n}$ units in which Δ=150 µs and the 180 ${}^{\circ }$ pulse was 91.6 µs .

The experimental parameters used for the acquisition of the various experiments on α -synuclein and CBP-ID4 are reported in Table 1. Spectra were calibrated using 2,2-dimethylsilapentane-5-sulfonic acid (DSS) as a reference for ${}^{1}H$ and ${}^{13}C$ ; ${}^{15}N$ was calibrated indirectly (Markley et al., 1998).

**Table 1:** Experimental parameters used.

		Dimension of acquired data			Spectral width (ppm)			NS ${}^{a}$	$d_{1}$ (s) ${}^{b}$
		t1	t2	t3	F1	F2	F3		
Experiments with α -synuclein									
${}^{1}H$ detected	${}^{1}H{-}^{15}N$ HSQC	800 ( ${}^{15}N$ )	2048 ( ${}^{1}H$ )		28.1	15.0		2	1.0
${}^{13}C$ detected	CON	512 ( ${}^{15}N$ )	1024 ( ${}^{13}C$ )		32.0	31.0		2	1.6
	CON ${}^{\mathrm{Pro}}$	128 ( ${}^{15}N$ )	1024 ( ${}^{13}C$ )		5.0	31.0		2	1.6
	$(H){\mathrm{CBCACON}}^{\mathrm{Pro}}$	128 ( ${}^{13}C$ )	32 ( ${}^{15}N$ )	1024 ( ${}^{13}C$ )	60.0	5.0	30.0	4	1.0
	$(H){\mathrm{CCCON}}^{\mathrm{Pro}}$	128 ( ${}^{13}C$ )	32 ( ${}^{15}N$ )	1024 ( ${}^{13}C$ )	70.0	5.0	30.0	4	1.0
	$(H){\mathrm{CBCANCO}}^{\mathrm{Pro}}$	128 ( ${}^{13}C$ )	16 ( ${}^{15}N$ )	1024 ( ${}^{13}C$ )	60.0	5.0	30.0	8	1.0
${}^{1}H$ and ${}^{13}C$ detected	CON/HN	600 ( ${}^{15}N$ )	1024 ( ${}^{13}C$ )		35.0	31.0		2	1.6
(using multiple receivers)		600 ( ${}^{15}N$ )	2048 ( ${}^{1}H$ )		35.0	15.0		4	
Experiments with CBP-ID4									
${}^{1}H$ detected	${}^{1}H{-}^{15}N$ HSQC	800 ( ${}^{15}N$ )	2048 ( ${}^{1}H$ )		30.0	15.0		2	1.0
${}^{13}C$ detected	CON	1024 ( ${}^{15}N$ )	1024 ( ${}^{13}C$ )		38.0	30.0		2	2.0
	CON ${}^{\mathrm{Pro}}$	170 ( ${}^{15}N$ )	1024 ( ${}^{13}C$ )		6.5	30.0		2	2.0
	$(H){\mathrm{CBCACON}}^{\mathrm{Pro}}$	128 ( ${}^{13}C$ )	64 ( ${}^{15}N$ )	1024 ( ${}^{13}C$ )	64.5	6.5	30.0	4	1.0
	$(H){\mathrm{CCCON}}^{\mathrm{Pro}}$	128 ( ${}^{13}C$ )	64 ( ${}^{15}N$ )	1024 ( ${}^{13}C$ )	75.7	6.5	30.0	4	1.0
	$(H){\mathrm{CBCANCO}}^{\mathrm{Pro}}$	128 ( ${}^{13}C$ )	22 ( ${}^{15}N$ )	1024 ( ${}^{13}C$ )	64.5	6.5	30.0	16	1.0
	$(\mathrm{HCA}){\mathrm{COCON}}^{\mathrm{Pro}}$	96 ( ${}^{13}C$ )	64 ( ${}^{15}N$ )	1024 ( ${}^{13}C$ )	10.8	6.5	30.0	8	1.5

${}^{a}$ Number of acquired scans. ${}^{b}$ Inter-scan delay.

## 3 Results and discussion

### 3.1 Advantages of focusing on proline residues

**Figure 1:**
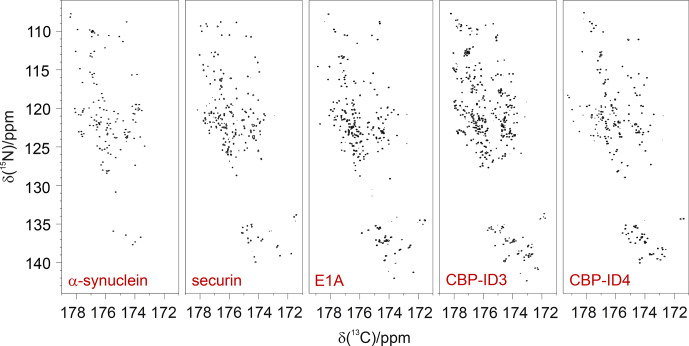
Proline residues are abundant in IDPs/IDRs, and their ${}^{15}N$ resonances can be easily detected. They fall in a specific, isolated region of the 2D CON spectrum, as illustrated by the examples in the figure. From left to right: α -synuclein (140 aa, 4 % Pro; Bermel et al., 2006b), human securin (200 aa, 11 % Pro; Bermel et al., 2009), E1A (243 aa, 16 % Pro; Hošek et al., 2016), CBP-ID3 (407 aa 18 % Pro; Contreras-Martos et al., 2017), and CBP-ID4 (207 aa, 22 % Pro; Piai et al., 2016).

In highly flexible and disordered proteins, contributions to signals' chemical shifts deriving from the local environment are averaged out, leaving mainly those contributions due to the covalent structure of the polypeptide. Chemical shift ranges predicted for ${}^{15}N$ resonances of imino acids such as proline are quite different from those predicted for amino acids, which is expected from the different chemical structure. The 2D CON spectra of several disordered proteins of different size and sequence complexity, reported in Fig. 1, clearly show that proline residues are quite abundant in IDPs/IDRs, and that ${}^{15}N$ resonances of proline residues indeed fall in a well-isolated spectral region.

**Figure 2:**
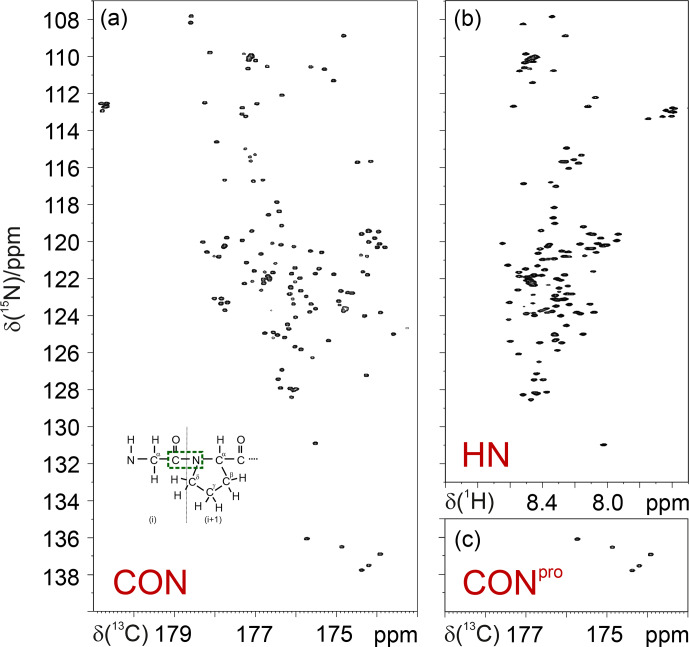
Comparison of the 2D CON **(a)** and 2D HN **(b)** spectra recorded on α -synuclein. The CON ${}^{\mathrm{pro}}$ spectrum **(c)**, shown below the HN panel, clearly illustrates how this experiment provides the missing information with respect to that available in the HN-detected spectrum. In the inset of the 2D CON spectrum, a scheme of a Gly ${}_{i-1}-{\text{Pro}}_{i}$ dipeptide highlights the nuclei that give rise to the ${C^{\prime }}_{i-1}-N_{i}$ correlations detected in CON spectra (in green).

Thus, ${}^{15}N$ resonances of proline residues in IDPs/IDRs can be selectively irradiated, enabling us to focus on this spectral region. This can be achieved through the use of band-selective ${}^{15}N$ pulses, as shown for the simple case of the CON experiment (Murrali et al., 2018). The selective CON spectrum in the proline region (CON ${}^{\mathrm{Pro}}$ ; Fig. 2) provides the complementary information that is missing in 2D HN correlation experiments, even when pH and temperature are optimized to enhance the detectability of amide protons (Fig. 2).

The same strategy that exploits band-selective ${}^{15}N$ pulses can be used to design experimental variants of triple resonance ${}^{13}C$ -detected experiments to focus on the ${}^{15}N$ proline region and enable us to selectively detect the desired correlations. When implementing this idea into these experiments, such as the 3D (H)CBCACON (Bermel et al., 2009), ${}^{15}N$ pulses could all be substituted with band-selective ones. However, instead of substituting all ${}^{15}N$ pulses, it is sufficient to introduce a 180 ${}^{\circ }$ band-selective ${}^{15}N$ pulse in one of the ${C^{\prime }}_{i-1}-N_{i}$ coherence transfer steps to introduce the desired selectivity in the proline region.

**Figure 3:**
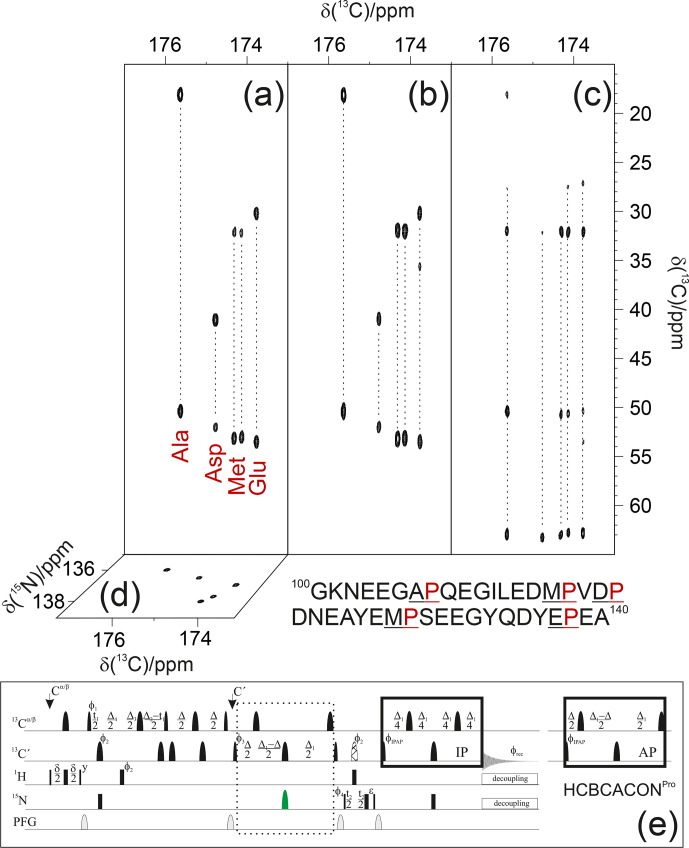
The implementation of the proposed strategy on α -synuclein renders NMR spectra so informative that proline resonances can be assigned just by visual inspection of the figure. To this end, the first ${}^{13}C{-}^{13}C$ planes of the 3D $(H){\mathrm{CBCACON}}^{\mathrm{Pro}}$ **(a)**, 3D $(H){\mathrm{CCCON}}^{\mathrm{Pro}}$ **(b)**, and 3D $(H){\mathrm{CBCANCO}}^{\mathrm{Pro}}$ **(c)** are shown, and the ${}^{13}C{-}^{15}N$ plane of the 3D $(H){\mathrm{CBCACON}}^{\mathrm{Pro}}$ is also shown **(d)**. The portion of the primary sequence of α -synuclein hosting its five proline residues is also reported (below the spectra). The pulse sequence for acquiring the 3D $(H){\mathrm{CBCACON}}^{\mathrm{Pro}}$ experiment **(e)** is reported as an example of the implementation of the proposed approach (dotted box). The delays are $\varepsilon =t_{2}(0)$ , δ=3.6 ms, Δ=9 ms, $\Delta_{1}=25$ ms, $\Delta_{2}=8$ ms, $\Delta_{3}=\Delta_{2}$ – $\Delta_{4}=5.8$ ms, and $\Delta_{4}=2.2$ ms. The phase cycle is as follows: $\phi_{1}=$ x, -x; $\phi_{2}=$ 8(x), 8(-x); $\phi_{3}=$ 4(x), 4(-x); $\phi_{4}=$ 2(x), 2(-x); $\phi_{\mathrm{IPAP}}(\text{IP})=$ x; and $\phi_{\mathrm{IPAP}}(\text{AP})=$ -y; $\phi_{\mathrm{rec}}=$ x, -x, -x, x, -x, x, x, -x. Quadrature detection was obtained by incrementing phase $\phi_{3}(t_{1})$ and $\phi_{4}(t_{2})$ in a States–TPPI (time-proportional phase incrementation) manner. The IPAP (in-phase/antiphase) approach was implemented for homonuclear decoupling in the direct acquisition dimension to suppress the large one bond scalar coupling constants ( ${}^{1}J_{C\alpha -C^{\prime }}$ ; Felli and Pierattelli, 2015); alternative approaches can be implemented that exploit band-selective homonuclear decoupling (Alik et al., 2020; Ying et al., 2014) or processing algorithms that, thus, only require the in-phase spectra (Karunanithy and Hansen, 2021; Shimba et al., 2003).

As an example, the pulse sequence of the 3D $(H){\mathrm{CBCACON}}^{\mathrm{Pro}}$ experiment is shown in Fig. 3. The inclusion of the ${}^{15}N$ band-selective pulse in the $C^{\prime }-N$ coherence transfer step is used to generate the ${C^{\prime }}_{i-1}-N_{i}$ antiphase coherence ( ${2C^{\prime }}_{y}N_{z}$ ) involving the ${}^{15}N$ nuclear spin of proline residues ( i ); for all other amino acid types, the evolution of the ${C^{\prime }}_{i-1}-N_{i}$ scalar coupling ( ${}^{1}J_{{C^{\prime }}_{i-1}-N_{i}}$ ) is refocused by the 180 ${}^{\circ }$ band-selective pulse on the carbonyl carbon nuclei only. To achieve the desired selectivity on the ${}^{15}N$ proline resonances with respect to those of all other amino acids, an 8 ms RE-BURP pulse (Geen and Freeman, 1991) was used here; this pulse may appear quite long, but it can be accommodated well in the $C^{\prime }-N$ coherence transfer block that requires about 32 ms ( $1/2^{1}J_{{C^{\prime }}_{i-1}-N_{i}}$ ). Considering an 8–10 ppm spectral width necessary to cover the ${}^{15}N$ proline region in the indirect dimension (Fig. 1), the implementation of this ${}^{15}N$ band-selective pulse allows us to reduce the spectral width by a factor of about 4 with respect to that needed to cover the whole spectral region in which backbone ${}^{15}N$ nuclear spins resonate, i.e. about 36–40 ppm. This means that the same resolution can be achieved in a fraction of the time since one-quarter (or less) of the free induction decays (FIDs) should be collected, provided sensitivity is not a limiting factor. Thus, it becomes feasible to acquire spectra with very high resolution, extending the acquisition time in all the indirect dimensions to contrast with the reduced chemical shift dispersion typical of IDPs. Non-uniform sampling strategies (Hoch et al., 2014; Kazimierczuk et al., 2010, 2011; Robson et al., 2019) can, of course, be implemented to reduce acquisition times; also, in this case, reducing the spectral complexity (the number of cross-peaks is reduced when focusing on proline ${}^{15}N$ resonances only) is expected to contribute to a reduction in experimental times. Out of the full 3D spectrum, only a small portion, the one containing the information that is completely missing in amide proton detected experiments, can thus be acquired with the necessary resolution to provide site-specific atomic information.

Since $C^{\prime }$ detected experiments all exploit the ${C^{\prime }}_{i-1}-N_{i}$ correlation, which is an inter-residue correlation linking the nitrogen of an amino acid ( i ) to the carbonyl carbon of the previous one ( i-1 ) across the peptide bond (Fig. 2 inset), focusing on proline residues can facilitate the identification of specific $X_{i-1}-{\text{Pro}}_{i}$ pairs through an inspection of $C^{\alpha }$ and $C^{\beta }$ chemical shifts of the amino acid preceding the proline residues. Such information can be achieved through the 3D $(H){\mathrm{CBCACON}}^{\mathrm{Pro}}$ experiment and can be very useful for identifying specific pairs such as Gly/Pro, Ala/Pro, Ser/Pro, and Thr/Pro. Acquisition of the 3D $(H){\mathrm{CCCON}}^{\mathrm{Pro}}$ experiment, in parallel to the 3D $(H){\mathrm{CBCACON}}^{\mathrm{Pro}}$ , provides information on aliphatic ${}^{13}C$ nuclear chemical shifts of the whole side chain. This contributes to narrowing down the possibilities in all cases in which it is not possible to identify the type of amino acid preceding the proline by considering only their ${}^{13}C^{\alpha }$ and ${}^{13}C^{\beta }$ chemical shifts. This is the case for the example of Arg/Lys/Gln or Phe/Leu.

Similarly, the insertion of the ${}^{15}N$ band-selective pulse for the proline region in the 3D (H)CBCANCO (Bermel et al., 2006a) enables us to detect the ${}^{13}C$ resonances of the whole proline ring, providing complementary information for the sequence-specific assignment. The closed proline ring introduces an additional heteronuclear scalar coupling ( ${}^{1}J_{N_{i}-C\delta i}$ ) that also provides the correlations with ${}^{13}C^{\delta }$ and ${}^{13}C^{\gamma }$ , which is parallel to the ${}^{13}C^{\alpha }$ and ${}^{13}C^{\beta }$ chemical shifts. Indeed, the band-selective pulses used for the ${}^{13}C$ aliphatic region also cover ${}^{13}C^{\gamma }$ and ${}^{13}C^{\delta }$ resonances (not only ${}^{13}C^{\alpha }$ and ${}^{13}C^{\beta }$ ones). In addition, analysis of the observed chemical shifts for proline side chains can be correlated to the local conformation, in particular to the *cis*/trans isomers of the peptide bond involving proline nitrogen nuclei (Schubert et al., 2002; Shen and Bax, 2010). Finally, additional information for sequence-specific assignment can be achieved by exploiting the same approach for the COCON experiment in its ${}^{13}C$ start (Bermel et al., 2006b; Felli et al., 2009) as well and in its ${}^{1}H$ start variants (Mateos et al., 2020).

### 3.2 Assignment strategy

To illustrate the approach, experiments were acquired on the well-known IDP α -synuclein. Even if this protein only contains a small number of proline residues ( 5 out of 140 ), they are all clustered in a small portion of it (108–138) and, thus, constitute about 15 % of the amino acids in this region. Furthermore, this terminus has a very peculiar amino acidic composition (36 % Asp/Glu , 9 % Tyr), and it was shown to be the part of the protein that is involved in sensing calcium concentration jumps associated with the transmission of nerve signals (Binolfi et al., 2006; Lautenschläger et al., 2018; Nielsen et al., 2001). Proline residues in between two negatively charged amino acids (Asp-Pro-Asp and Glu-Pro-Glu) were shown to facilitate the interaction of carboxylate side chains of Asp and Glu with calcium, even in a flexible and disordered state (Pontoriero et al., 2020).

Focusing on the proline ${}^{15}N$ region in case of α -synuclein greatly simplifies the spectral complexity, enabling us to illustrate the sequence-specific assignment of the resonances just by visual inspection of the first planes of the 3D spectra described here (Fig. 3).

Indeed, the $C^{\prime }-N$ projections of the 3D spectra ( ${}^{13}C{-}^{15}N$ planes) show that the cross-peaks are well resolved in both dimensions; the selection of the ${}^{15}N$ proline region enables us to differentiate the signals through the carbonyl carbon chemical shifts of the preceding amino acid. Therefore, an inspection of the first ${}^{13}C{-}^{13}C$ plane of the 3D $(H){\mathrm{CBCACON}}^{\mathrm{Pro}}$ experiment (Fig. 3a) shows the distinctive ${}^{13}C^{\alpha }$ and ${}^{13}C^{\beta }$ chemical shift patterns of the residues preceding proline that, by comparison with the primary sequence of the protein, already suggests the identity of three residue pairs to us, i.e. Ala–Pro, Asp–Pro, and Glu–Pro. These can, thus, be assigned to Ala 107–Pro 108, Asp 119–Pro 120, Glu 138–Pro 139. Comparison with the first ${}^{13}C{-}^{13}C$ plane of the 3D $(H){\mathrm{CCCON}}^{\mathrm{Pro}}$ (Fig. 3b) confirms that an extra cross-peak can be detected for the Glu–Pro pair, which is as expected for amino acids that have a side chain with more than two aliphatic carbon atoms. The remaining signals derive from the two Met–Pro pairs, in agreement with the observed chemical shifts. They can be assigned in a sequence-specific manner by comparing these spectra with the complementary ones based on amide proton detection. The final panel shows the first ${}^{13}C{-}^{13}C$ plane of the 3D $(H){\mathrm{CBCANCO}}^{\mathrm{Pro}}$ (Fig. 3c). This experiment reveals the correlations of the ${}^{15}N$ with ${}^{13}C^{\alpha }$ and ${}^{13}C^{\beta }$ within each amino acid. In the case of proline, the closed ring introduces additional scalar couplings that are responsible for two additional cross-peaks, i.e. the ones of the ${}^{15}N$ with ${}^{13}C^{\gamma }$ and ${}^{13}C^{\delta }$ , as clearly observed in Fig. 3. Chemical shifts show only minor differences between the resonances, but these are still significant to discriminate between the different residues, provided spectra are acquired with high resolution.

### 3.3 A challenging case

**Figure 4:**
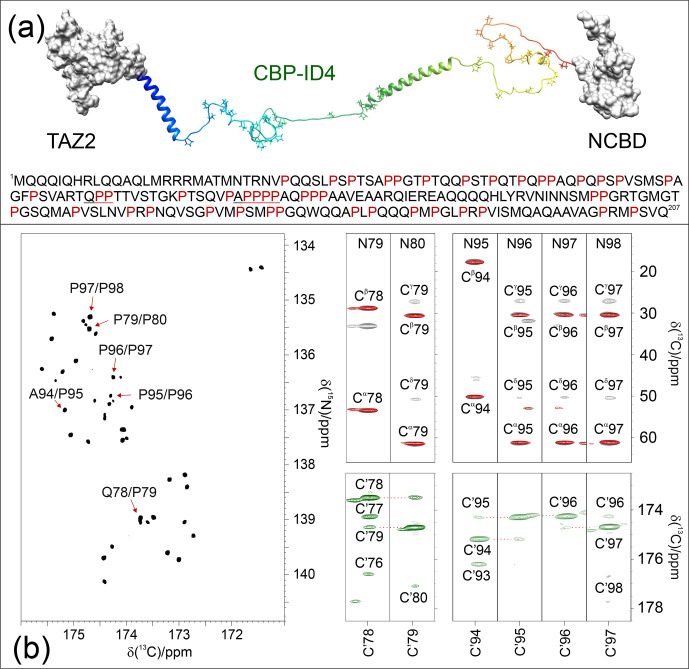
The case of CBP-ID4 (residues 1851–2057 of human CBP). **(a)** One of the possible conformations of the CBP-ID4 fragment (blue to red ribbons) and the two flanking domains, TAZ2 and NCBD, clearly demonstrate that, in this region of CBP, the intrinsically disordered part is highly prevalent with respect to ordered ones. The amino acid sequence of CBP-ID4 is also reported. **(b)** The 2D CON ${}^{\mathrm{Pro}}$ spectrum shown on the left, which, at first sight, could seem like a 2D spectrum of a small globular protein, reports the proline fingerprint of this complex IDR. Several strips extracted from the 3D $(H){\mathrm{CBCACON}}^{\mathrm{Pro}}$ (grey contours), 3D $(H){\mathrm{CCCON}}^{\mathrm{Pro}}$ (red contours), and 3D $(\mathrm{HCA}){\mathrm{COCON}}^{\mathrm{Pro}}$ (green contours) are shown to illustrate the information available for the sequence-specific resonance assignment of this proline-rich fragment of CBP-ID4.

A compelling example of complexity is provided by the fourth flexible linker (ID4) of CREB-binding protein (CBP), a large transcription co-regulator (Dyson and Wright, 2016). CBP-ID4 connects two well-characterized globular domains (TAZ2, 92 amino acids, and NCBD, 59 amino acids) (De Guzman et al., 2000; Kjaergaard et al., 2010) and is constituted by 207 amino acids out of which 45 are proline residues, including several repeated PP motifs (Piai et al., 2016) (Fig. 4a). The 2D CON ${}^{\mathrm{Pro}}$ spectrum of CBP-ID4, reported in Fig. 4b (left panel), shows the ${C^{\prime }}_{i-1}-N_{i}$ correlations of proline residues of ID4. Interestingly, despite the small spectral region, a high number of resolved resonances is observed. The initial count of cross-peaks in this spectrum reveals 42 out of the 45 expected correlations, highlighting the potential of this experimental strategy for the investigation of IDRs/IDPs of increasing complexity. The excellent chemical shift dispersion of the inter-residue ${C^{\prime }}_{i-1}-N_{i}$ correlations is certainly one of the most important aspects for reducing cross-peak overlap. The resolution is further enhanced in this region by the narrow linewidths of proline ${}^{15}N$ resonances due to the lack of the dipolar contribution of an amide proton to the transverse relaxation.

These two features contribute to establishing this spectral region as a key one for the assignment of a large IDR. Indeed, when passing from 2D to 3D experiments, long acquisition times in the ${}^{15}N$ dimension are possible and enable us to provide the extra contribution to the resolution enhancement needed to focus on complex IDRs and to collect additional information on the proline residues and their neighbouring amino acids. As an example of the quality of the spectra, Fig. 4b reports several strips extracted from the proline selective 3D experiments that were essential for the investigation of a particularly proline-rich region of ID4, the one in between two partially populated α -helices (Piai et al., 2016). This is composed of 27 prolines (out of 76 amino acids) which constitute 35 % of the amino acids in this region, including several proline-rich motifs (PXP, PXXP, and PP, as well as PPP and PPPP, where P stands for a proline and X for any other amino acid). Figure 4b shows the strips, extracted from the 3D proline selective experiments that were used to assign resonances in the two proline-rich regions (78–80 and 94–98). The strips extracted from the 3D $(H){\mathrm{CBCACON}}^{\mathrm{Pro}}$ and 3D $(H){\mathrm{CCCON}}^{\mathrm{Pro}}$ (Fig. 4b; right panels; black and red contours respectively) are very useful for identifying the $X_{i-1}-{\text{Pro}}_{i}$ pairs that match, in this case, with a Gln–Pro and an Ala–Pro pair, as well as several Pro–Pro ones. The 3D $(H){\mathrm{CBCANCO}}^{\mathrm{Pro}}$ completes the picture by providing information about ${}^{13}C$ resonances of each proline ring ( $C^{\alpha }$ , $C^{\beta }$ , $C^{\gamma }$ , and $C^{\delta }$ ; not shown for sake of clarity in the figure). However, in regions with a high abundance of proline residues, additional information is needed for their sequence-specific assignment. To this end, the 3D $(\mathrm{HCA}){\mathrm{COCON}}^{\mathrm{Pro}}$ (Fig. 4b; right panels; green contours) is very useful, as demonstrated for these two proline-rich fragments. This experiment, which includes an isotropic mixing element in the carbonyl region (MOCCA, in this case; Felli et al., 2009; Furrer et al., 2004) enables us to detect correlations of a carbonyl with the neighbouring ones through the small ${}^{3}J_{C^{\prime }C^{\prime }}$ scalar couplings. In case of proline residues, the most intense cross-peak is generally observed for the preceding amino acid ( ${C^{\prime }}_{i-2}$ ). However, additional peaks are also detected with neighbouring ones and support the sequence-specific assignment process.

It is interesting to note that, once the sequence-specific assignment becomes available, ${C^{\prime }}_{i-1}-N_{i}$ correlations fall in distinctive spectral regions of the 2D CON ${}^{\mathrm{Pro}}$ spectrum, as already pointed out for selected residue pairs such as Gly–Pro, Ser–Pro, Thr–Pro, and Val–Pro (Murrali et al., 2018). For example, an inspection of Fig. 1 allows us to identify Gly–Pro pairs in all the CON spectra of different proteins from their characteristic chemical shifts (in the top-right portion of the proline region). An additional contribution towards smaller ${}^{15}N$ chemical shifts can also be identified in cases in which more than one proline follows a specific amino-acid-type, such as for Ala–Pro, Met–Pro, and Gln–Pro cross-peaks that are shifted to lower ${}^{15}N$ chemical shifts when an additional proline follows in the primary sequence. These effects likely originate from a combination of effects deriving from the covalent structure (primary sequence in this case) and from local conformations. Needless to say, the experimental investigation of these aspects in more detail constitutes an important point that describes the structural and dynamic properties at the atomic resolution of the proline-rich parts of highly flexible IDRs. The proposed experiments are, thus, expected to become of general applicability for studies of IDPs/IDRs in solution.

The data generated on proline-rich sequences are, of course, very relevant to populate databases such as the Biological Magnetic Resonance Data Bank (BMRB, https://bmrb.io/, last access: 17 June 2021), with information on proline residues in highly flexible protein regions. This, in turn, will generate more accurate reference data in chemical shift databases to determine local structural propensities through the comparison of experimental shifts with reference ones (Camilloni et al., 2012; Tamiola et al., 2010), thus improving our understanding of the importance of transient secondary structure elements in determining protein function. In this respect, CBP itself provides another enlightening example with the third disordered linker of CBP (CBP-ID3; residues 674-1079 of CBP), which features a high number of proline residues (75 out of 406 residues) representing 18 % of its primary sequence. The distribution, in this case, is along the entire sequence but less frequent toward the end, where a β strand conformation propensity is sampled (Contreras-Martos et al., 2017). Also, in this case, the distribution of proline residues is important for shaping the conformational space accessible to the polypeptide, facilitating the interaction with protein's partners. Determination of additional observables, such as the ${}^{3}J_{C^{\prime }C^{\prime }}$ through the 3D $(\mathrm{HCA}){\mathrm{COCON}}^{\mathrm{Pro}}$ experiment or of different ones through modified experimental variants of these experiments, are expected to contribute to the characterization of novel motifs in IDRs/IDPs.

The experimental strategy proposed here focuses on a remarkably small spectral region which, however, turns out to be one of the most interesting ones, in particular from the perspective of studying IDPs/IDRs of increasing complexity, somehow reminiscent of other strategies that have been proposed in which only selected residue types are investigated to access information on challenging systems (such as, for example, the studies of large globular proteins enabled by methyl transverse-relaxation-optimized spectroscopy (methyl​-TROSY) spectroscopy; Kay, 2011; Schütz and Sprangers, 2020). Interestingly, the analysis of the NMR spectra presented here enables one to classify the observed cross-peaks into residue types, also in absence of a sequence-specific assignment. This might provide interesting information for complex IDPs/IDRs in which one is interested in the investigation of the contribution of specific residue types, such as to monitor the occurrence of post-translational modifications or even other phenomena that are more difficult to investigate like liquid–liquid-phase separation.

## 4 Conclusions and perspectives

Detection and assignment of proline-rich regions of highly flexible intrinsically disordered proteins allows us to have a glimpse of the ways in which proline residues encode specific properties in IDRs/IDPs by simply tuning their distribution along the primary sequence. NMR spectroscopy is particularly well suited for the task, since proline residues have attractive features from the NMR point of view, starting from the peculiar chemical shifts of ${}^{15}N$ nuclear spins. In addition, the lack of the attached amide proton implies that one of the major contributions to relaxation of ${}^{15}N$ spins is absent and, thus, proline nitrogen signals have small linewidths. These characteristics make them a very useful starting point for sequential assignment purposes and structure characterization. Furthermore, they provide a set of NMR signals with promising properties to enable high-resolution studies of increasingly large IDPs/IDRs.

Several approaches, either based on $H^{N}$ or on $H^{\alpha }$ direct detection, have been proposed to bypass the problem introduced in the sequence-specific assignment by the lack of amide protons typical of proline residues (Hellman et al., 2014; Kanelis et al., 2000; Karjalainen et al., 2020; Löhr et al., 2000; Mäntylahti et al., 2010; Tossavainen et al., 2020; Wong et al., 2018). While these results can be useful for systems with moderate complexity ( $H^{\alpha }$ detection) or for systems that feature isolated proline residues in the primary sequence ( $H^{N}/H^{\alpha }$ ), they are not as efficient for complex IDRs/IDPs in which high resolution is mandatory and in which consecutive proline residues are often encountered. Detection of ${}^{15}N$ -based experiments can also provide direct observation of proline nuclei (Chhabra et al., 2018), but despite the excellent resolution achievable with IDPs, these experiments still suffer from sensitivity limitations.

To conclude, the experiments proposed here are crucial to assign intrinsically disordered protein regions presenting many repeated motifs, including proline residues and poly-proline segments, to reduce spectral complexity and experimental time without compromising resolution. Since NMR probeheads with high ${}^{13}C$ sensitivity have become widely available, it is expected that this set of experiments will be applied as an easy-to-use tool that will also complement the $H^{N}$ -based assignment.

## Supplement

10.5194/mr-2-511-2021-supplement The supplement related to this article is available online at: https://doi.org/10.5194/mr-2-511-2021-supplement.

## Data Availability

Data are available from the authors upon request.

## Code availability

The codes of the pulse sequences used are provided in the Supplement.
