# Supplementary material for: Exclusively heteronuclear NMR experiments for the investigation of intrinsically disordered proteins: focusing on proline residues
Source: Magn Reson (Gott). 2021 Jul 1;2(1):511–22. doi: 10.5194/mr-2-511-2021 (PMC10539766; doi:10.5194/mr-2-511-2021)
Supplement: The supplement related to this article is available online at: https://doi.org/10.5194/mr-2-511-2021-supplement. [file mr-2-511-supplement.pdf]

Supplement of Magn. Reson., 2, 511–522, 2021
https://doi.org/10.5194/mr-2-511-2021-supplement
© Author(s) 2021. CC BY 4.0 License.

Supplement of

Exclusively heteronuclear NMR experiments for the investigation of intrinsically disordered proteins: focusing on proline residues

Isabella C. Felli et al.

Correspondence to: Isabella C. Felli (felli@cerm.unifi.it) and Roberta Pierattelli (roberta.pierattelli@unifi.it)

The copyright of individual parts of the supplement might differ from the article licence.

Supplementary information

The pulse sequences used to acquire the 3D experiments to focus on proline residues are reported hereafter:

Pulse sequence 1 – **3D (H)CBCACON_pro**

Pulse sequence 2 – **3D (H)CCCON_pro**

Pulse sequence 3 – **3D (H)CBCANCO_pro**

Pulse sequence 4 – **3D (H)CACOCON_pro**

Pulse sequence 1 – **3D (H)CBCACON_pro**

```
;c_hcbcacon_ia3d_pro
;avance-version (20/07/28)
;(H)CbCaCON
;3D sequence with
;   13C detected correlation for triple resonance using
;      multiple inept transfer steps
;
;      F2(Ha/b) -> F1(Ca/b,t1) -> F1(Ca) -> F1(C=O)
;              -> F3(N,t2) -> F1(C=O,t3)
;
;on/off resonance 13C pulses using shaped pulses
;phase sensitive (t1)
;phase sensitive (t2)
;using constant time in t1
;using IPAP scheme for virtual decoupling
;using selective N pulse for Pro
;(use parameterset )
;
;M.G. Murrali, A. Piai, W. Bermel, I.C. Felli & R. Pierattelli,
;   ChemBioChem 19, 1625-1629 (2018)
;W. Bermel, I. Bertini, V. Csizmok, I. C. Felli, R. Pierattelli &
;   P. Tompa, J. Magn. Reson. 198, 275-281 (2009)
;(W. Bermel, I. Bertini, I.C. Felli, R. Kuemmerle
;   & R. Pierattelli, J. Magn. Reson. 178, 56-64 (2006) )
;(W. Bermel, I. Bertini,L. Duma, I.C. Felli, L. Emsley,  R. Pierattelli,
;   P.R. Vasos, Angew. Chem. Int. Ed. 44, 3089-3092 (2005) )
;(L. Duma, S. Hediger, A. Lesage & L. Emsley,
;   J. Magn. Reson. 164, 187-195 (2003) )
;
;$CLASS=HighRes
;$DIM=3D
;$TYPE=
;$SUBTYPE=
;$COMMENT=


prosol relations=<triple_c>


#include <Avance.incl>
#include <Grad.incl>
#include <Delay.incl>


"p4=p3*2"
```

```
"p22=p21*2"
"d11=30m"
"d12=20u"

"d3=1.1m"
"d4=1.8m"
"d22=4.5m"
"d23=16.6m"
"d25=4.0m"


"d0=3u"
"d10=3u"
"d20=d25-p12-4u"

"in0=inf1/2"
"in10=inf2/2"

"in20=in0"

"td1=tdmax(td1,d20*2,in20)"


"DELTA=d10*2+larger(p4,p8)"
"DELTA1=d25-d0-larger(p12,p22)-d3-p4"
"DELTA2=d23-d22-p12"
"DELTA3=d23-p12-larger(p12,p59)/2-4u"
"DELTA4=d23/2-p12/2"
"DELTA5=d25-p12-4u"
"DELTA6=d23-d22-p12-larger(p12,p59)/2"


"l0=1"


"spoff13=bf1*((cnst22/2-cnst21/2)/1000000)"
"spoff23=0"
"spoff24=0"
"spoff25=0"
"spoff26=bf1*((cnst21-cnst23)/1000000)"
"spoff27=bf1*((cnst22-cnst21)/1000000)"

"o1_F1=bf1*cnst23/1000000"

aqseq 321


1 ze
```

```
  d11 pl12:f2 pl16:f3
2 d11 do:f2 do:f3
3 d1 fq=cnst23(bf ppm):f1
  d12 pl2:f2 pl3:f3
  50u UNBLKGRAD
  (p3 ph1):f2
  d4
  (center (p12:sp24 ph1) (p4 ph1):f2 )
  d4
  (p3 ph2):f2
  p16:gp1
  d16
  (p11:sp23 ph3)
  d0
  (center (p12:sp26 ph6) (p22 ph1):f3 )
  d3
  (p4 ph6):f2
  DELTA1
  (p12:sp24 ph1)
  d20
  (p12:sp26 ph1)
  4u
  (p11:sp25 ph1)
  4u
  (p12:sp26 ph1)
  DELTA5
  (p12:sp24 ph1)
  4u
  (p12:sp26 ph1)
  DELTA5
  (p11:sp23 ph1)
  p16:gp2
  d16 fq=cnst21(bf ppm):f1
  (p11:sp23 ph5)
  d22
  (p12:sp27 ph1)
  DELTA6
  (center (p12:sp24 ph1) (p59:sp46 ph1):f3 )
  DELTA3
  (p12:sp27 ph1)
  4u
  (p11:sp25 ph1)
```

```
  p16:gp3
  d16 pl3:f3

  (p21 ph4):f3
  d10
  (center (p8:sp13 ph6) (p4 ph6):f2 )
  d10
  (p22 ph1):f3
  DELTA
  (p21 ph1):f3

  p16:gp4
  d16 pl12:f2

  if "l0 %2 == 1"
    {
    (p11:sp23 ph1)
    DELTA4
    (p12:sp27 ph1)
    DELTA4
    (center (p12:sp24 ph1) (p22 ph1):f3 )
    DELTA4
    (p12:sp27 ph1)
    DELTA4 pl16:f3
    }
  else
    {
    (p11:sp23 ph7)
    d22
    (p12:sp27 ph1)
    DELTA2
    (center (p12:sp24 ph1) (p22 ph1):f3 )
    DELTA4
    DELTA4 pl16:f3
    (p12:sp27 ph1)
    }

  4u BLKGRAD
  go=2 ph31 cpd2:f2 cpd3:f3
  d11 do:f2 do:f3 mc #0 to 2

#   ifdef LABEL_F1
    F1I(iu0, 2)
#   else
    F2I(iu0, 2)
#   endif /*LABEL_F1*/
```

```
    F1PH(calph(ph3, +90), caldel(d0, +in0) & caldel(d20, -in20))
    F2PH(calph(ph4, +90), caldel(d10, +in10))
exit

ph1=0
ph2=1
ph3=0 2
ph4=0 0 2 2
ph5=0 0 0 0 2 2 2 2
ph6=0 0 0 0 0 0 0 0 2 2 2 2 2 2 2 2
ph7=3
ph31=0 2 2 0 2 0 0 2

;pl1 : f1 channel - power level for pulse (default)
;pl2 : f2 channel - power level for pulse (default)
;pl3 : f3 channel - power level for pulse (default)
;pl12: f2 channel - power level for CPD/BB decoupling
;pl16: f3 channel - power level for CPD/BB decoupling
;sp13: f1 channel - shaped pulse 180 degree  (adiabatic)
;sp23: f1 channel - shaped pulse  90 degree  (on resonance)
;sp24: f1 channel - shaped pulse 180 degree  (on resonance)
;sp25: f1 channel - shaped pulse  90 degree  (on resonance)
;                 for time reversed pulse
;sp26: f1 channel - shaped pulse 180 degree  (C=O off resonance)
;sp27: f1 channel - shaped pulse 180 degree  (Ca off resonance)
;sp46: f3 channel - shaped pulse 180 degree  (N, selective for Pro)
;p3 : f2 channel -  90 degree high power pulse
;p4 : f2 channel - 180 degree high power pulse
;p8 : f1 channel - 180 degree shaped pulse for inversion (adiabatic)
;p11: f1 channel -  90 degree shaped pulse
;p12: f1 channel - 180 degree shaped pulse
;p16: homospoil/gradient pulse                       [1 msec]
;p21: f3 channel -  90 degree high power pulse
;p22: f3 channel - 180 degree high power pulse
;p59: f3 channel - 180 degree shaped pulse (N, selective for Pro)
;d0 : incremented delay (F1 in 3D)                   [3 usec]
;d1 : relaxation delay; 1-5 * T1
;d3 : 1/(6J(HCa))                                    [1.1 msec]
;d4 : 1/(4J(HCa))                                    [1.8 msec]
;d10: incremented delay (F2 in 3D)                   [3 usec]
;d11: delay for disk I/O                             [30 msec]
;d12: delay for power switching                      [20 usec]
;d16: delay for homospoil/gradient recovery
;d20: decremented delay (F1 in 3D) = d25+d0-p12-4u
;d22: 1/(4J(COCa))                                   [4.5 msec]
;d23: 1/(4J(NCO))                                    [16.6 msec]
```

```
;d25: 1/(8J'(CaCb))                    [4.0 msec]
;cnst21: CO chemical shift (offset, in ppm)
;cnst22: Calpha chemical shift (offset, in ppm)
;cnst23: Caliphatic chemical shift (offset, in ppm)
;o1p: CO chemical shift (cnst21)
;l0: flag to switch between inphase and antiphase
;in0: 1/(2 * SW(Ca/b)) =  DW(Ca/b)
;nd0: 2
;in10: 1/(2 * SW(N)) =  DW(N)
;nd10: 2
;in20: = in0
;ns: 16 * n
;ds: >= 32
;td1: number of experiments in F1
;td2: number of experiments in F2
;FnMODE: States-TPPI (or TPPI) in F1   td1 max = n * d20 / in20 (n = 4 or = 2 with LABEL_F2)
;FnMODE: States-TPPI (or TPPI) in F2
;cpd2: decoupling according to sequence defined by cpdprg2
;cpd3: decoupling according to sequence defined by cpdprg3
;pcpd2: f2 channel - 90 degree pulse for decoupling sequence
;pcpd3: f3 channel - 90 degree pulse for decoupling sequence


;for z-only gradients:
;gpz1: 50%
;gpz2: 30%
;gpz3: 19%
;gpz4: 13%

;use gradient files:
;gpnam1: SMSQ10.100
;gpnam2: SMSQ10.100
;gpnam3: SMSQ10.100
;gpnam4: SMSQ10.100


                              ;preprocessor-flags-start
;LABEL_F2: to do ipap in F2 start experiment with
;           option -DLABEL_F2 (eda: ZGOPTNS)
                              ;preprocessor-flags-end


;use AU-program splitcomb [ipap 2] (F1 or F2 with LABEL_F2) to process data



;$Id: $
```

Pulse sequence 2 – **3D (H)CCCON_pro**

```
;c_hcccon_ia3d_pro
;avance-version (20/07/28)
;(H)CC(CO)N
;3D sequence with
;   13C detected correlation for triple resonance using
;      multiple inept transfer steps and
;      C-C FLOPSY16 spinlock
;
;      F1(Cali,t1) -> F1(Ca) -> F1(C=O)
;                          -> F3(N,t2) -> F1(C=O,t3)
;
;on/off resonance 13C pulses using shaped pulses
;phase sensitive (t1)
;phase sensitive (t2)
;using IPAP scheme for virtual decoupling
;using selective N pulse for Pro
;(use parameterset )
;
;M.G. Murrali, A. Piai, W. Bermel, I.C. Felli & R. Pierattelli,
;   ChemBioChem 19, 1625-1629 (2018)
;W. Bermel, I. Bertini, I.C. Felli, R. Kuemmerle
;   & R. Pierattelli, J. Magn. Reson. 178, 56-64 (2006)
;
;$CLASS=HighRes
;$DIM=3D
;$TYPE=
;$SUBTYPE=
;$COMMENT=


prosol relations=<triple_c>


#include <Avance.incl>
#include <Delay.incl>
#include <Grad.incl>


"p4=p3*2"
"p22=p21*2"
"d11=30m"
"d12=20u"

"d3=0.95m"
```

```
"d4=1.8m"
"d22=4.5m"
"d23=16.6m"

"d0=3u"
"d10=3u"

"in0=inf1/2"
"in10=inf2/2"

"DELTA=d10*2+p8"
"DELTA1=d3/2"
"DELTA2=d3/2+p22+p4+d0*2"
"DELTA3=d23-d22-p12"
"DELTA4=d23-p12-larger(p12,p59)/2-4u"
"DELTA5=d23/2-p12/2"
"DELTA6=d23-d22-p12-larger(p12,p59)/2"

"FACTOR2=(d9/(p6*188.448))"
"l1=FACTOR2"

"l0=1"

"spoff13=bf1*((cnst22/2-cnst21/2)/1000000)"
"spoff22=bf1*((cnst21-cnst22)/1000000)"
"spoff23=0"
"spoff24=0"
"spoff25=0"
"spoff26=bf1*((cnst21-cnst23)/1000000)"
"spoff27=bf1*((cnst22-cnst21)/1000000)"
"spoff28=0"

"o1_F1=bf1*cnst23/1000000"

aqseq 321

1 ze
  d11 pl12:f2 pl16:f3
2 d11 do:f2 do:f3
3 d1 fq=cnst23(bf ppm):f1
```

```
 d12 pl2:f2 pl3:f3
 50u UNBLKGRAD

 (p3 ph1):f2
 d4
 (center (p12:sp24 ph1) (p4 ph1):f2 )
 d4
 (p3 ph8):f2

 (p11:sp23 ph3)
 d0
 (p22 ph1):f3
 DELTA1
 (p12:sp26 ph1)
 DELTA1
 (p4 ph1):f2
 d0
 (p12:sp24 ph1)
 DELTA2
 (p12:sp26 ph1)
 DELTA1
 (p11:sp25 ph1)

 p19:gp1
 d16 pl10:f1
                                        ;begin FLOPSY16
4 p6*0.511 ph11
 p6*1.067 ph12
 p6*1.822 ph13
 p6*1.767 ph14
 p6*1.444 ph15
 p6*1.767 ph14
 p6*1.822 ph13
 p6*1.067 ph12
 p6*0.511 ph11

 p6*0.511 ph21
 p6*1.067 ph22
 p6*1.822 ph23
 p6*1.767 ph24
 p6*1.444 ph25
 p6*1.767 ph24
 p6*1.822 ph23
 p6*1.067 ph22
 p6*0.511 ph21

 p6*0.511 ph21
 p6*1.067 ph22
```

```
p6*1.822 ph23
p6*1.767 ph24
p6*1.444 ph25
p6*1.767 ph24
p6*1.822 ph23
p6*1.067 ph22
p6*0.511 ph21

p6*0.511 ph11
p6*1.067 ph12
p6*1.822 ph13
p6*1.767 ph14
p6*1.444 ph15
p6*1.767 ph14
p6*1.822 ph13
p6*1.067 ph12
p6*0.511 ph11

p6*0.511 ph11
p6*1.067 ph12
p6*1.822 ph13
p6*1.767 ph14
p6*1.444 ph15
p6*1.767 ph14
p6*1.822 ph13
p6*1.067 ph12
p6*0.511 ph11

p6*0.511 ph11
p6*1.067 ph12
p6*1.822 ph13
p6*1.767 ph14
p6*1.444 ph15
p6*1.767 ph14
p6*1.822 ph13
p6*1.067 ph12
p6*0.511 ph11

p6*0.511 ph21
p6*1.067 ph22
p6*1.822 ph23
p6*1.767 ph24
p6*1.444 ph25
p6*1.767 ph24
p6*1.822 ph23
p6*1.067 ph22
p6*0.511 ph21
```

```
p6*0.511 ph21
p6*1.067 ph22
p6*1.822 ph23
p6*1.767 ph24
p6*1.444 ph25
p6*1.767 ph24
p6*1.822 ph23
p6*1.067 ph22
p6*0.511 ph21

p6*0.511 ph21
p6*1.067 ph22
p6*1.822 ph23
p6*1.767 ph24
p6*1.444 ph25
p6*1.767 ph24
p6*1.822 ph23
p6*1.067 ph22
p6*0.511 ph21

p6*0.511 ph11
p6*1.067 ph12
p6*1.822 ph13
p6*1.767 ph14
p6*1.444 ph15
p6*1.767 ph14
p6*1.822 ph13
p6*1.067 ph12
p6*0.511 ph11

p6*0.511 ph11
p6*1.067 ph12
p6*1.822 ph13
p6*1.767 ph14
p6*1.444 ph15
p6*1.767 ph14
p6*1.822 ph13
p6*1.067 ph12
p6*0.511 ph11

p6*0.511 ph21
p6*1.067 ph22
p6*1.822 ph23
p6*1.767 ph24
p6*1.444 ph25
p6*1.767 ph24
p6*1.822 ph23
p6*1.067 ph22
```

```
p6*0.511 ph21

p6*0.511 ph21
p6*1.067 ph22
p6*1.822 ph23
p6*1.767 ph24
p6*1.444 ph25
p6*1.767 ph24
p6*1.822 ph23
p6*1.067 ph22
p6*0.511 ph21

p6*0.511 ph21
p6*1.067 ph22
p6*1.822 ph23
p6*1.767 ph24
p6*1.444 ph25
p6*1.767 ph24
p6*1.822 ph23
p6*1.067 ph22
p6*0.511 ph21

p6*0.511 ph11
p6*1.067 ph12
p6*1.822 ph13
p6*1.767 ph14
p6*1.444 ph15
p6*1.767 ph14
p6*1.822 ph13
p6*1.067 ph12
p6*0.511 ph11

p6*0.511 ph11
p6*1.067 ph12
p6*1.822 ph13
p6*1.767 ph14
p6*1.444 ph15
p6*1.767 ph14
p6*1.822 ph13
p6*1.067 ph12
p6*0.511 ph11
lo to 4 times l1
                                                  ;end FLOPSY16

4u
p19:gp2
d16 fq=cnst22(bf ppm):f1
20u pl12:f2
```

```
20u cpd2:f2

(p11:sp23 ph1)
d22
(p12:sp22 ph1)
4u
(p25:sp28 ph1)
d22
(p12:sp22 ph1)
4u
(p11:sp25 ph2)

4u do:f2
p16:gp3
d16 fq=cnst21(bf ppm):f1
20u cpd2:f2

(p11:sp23 ph1)
d22
(p12:sp27 ph1)
DELTA6
(center (p12:sp24 ph1) (p59:sp46 ph1):f3 )
DELTA4
(p12:sp27 ph1)
4u
(p11:sp25 ph1)

4u do:f2
p16:gp4
d16 pl3:f3
20u cpd2:f2

(p21 ph4):f3
d10
(p8:sp13 ph6)
d10
(p22 ph1):f3
DELTA
(p21 ph5):f3

if "l0 %2 == 1"
  {
  (p11:sp23 ph1)
  DELTA5
  (p12:sp27 ph1)
  DELTA5
  (center (p12:sp24 ph1) (p22 ph1):f3 )
  DELTA5
```

```
     (p12:sp27 ph1)
     DELTA5 pl16:f3
     }
  else
     {
     (p11:sp23 ph7)
     d22
     (p12:sp27 ph1)
     DELTA3
     (center (p12:sp24 ph1) (p22 ph1):f3 )
     DELTA5
     DELTA5 pl16:f3
     (p12:sp27 ph1)
     }

  4u BLKGRAD
  go=2 ph31 cpd3:f3
  d11 do:f2 do:f3 mc #0 to 2

#   ifdef LABEL_F1
     F1I(iu0, 2)
#   else
     F2I(iu0, 2)
#   endif /*LABEL_F1*/

     F1PH(calph(ph3, +90), caldel(d0, +in0))
     F2PH(calph(ph4, +90), caldel(d10, +in10))
exit


ph1=0
ph2=1
ph3=1 3
ph4=0 0 0 0 2 2 2 2
ph5=0 0 0 0 0 0 0 0 2 2 2 2 2 2 2 2
ph6=0
ph7=3
ph8=1 1 3 3

ph11=(720) 0
ph12=(720) 90
ph13=(720) 135
ph14=(720) 630
ph15=(720) 45

ph21=(720) 360
ph22=(720) 450
ph23=(720) 495
```

```
ph24=(720) 270
ph25=(720) 405

ph31=0 2 2 0 2 0 0 2 2 0 0 2 0 2 2 0


;pl1 : f1 channel - power level for pulse (default)
;pl2 : f2 channel - power level for pulse (default)
;pl3 : f3 channel - power level for pulse (default)
;pl10: f1 channel - power level for TOCSY-spinlock
;pl12: f2 channel - power level for CPD/BB decoupling
;pl16: f3 channel - power level for CPD/BB decoupling
;sp13: f1 channel - shaped pulse 180 degree  (adiabatic)
;sp22: f1 channel - shaped pulse 180 degree  (C=O off resonance)
;sp23: f1 channel - shaped pulse  90 degree  (on resonance)
;sp24: f1 channel - shaped pulse 180 degree  (on resonance)
;sp25: f1 channel - shaped pulse  90 degree  (on resonance)
;              for time reversed pulse
;sp26: f1 channel - shaped pulse 180 degree  (C=O off resonance)
;sp27: f1 channel - shaped pulse 180 degree  (Ca off resonance)
;sp28: f1 channel - shaped pulse 180 degree  (Ca on resonance)
;              sp28 requires higher selectivity than sp24
;sp46: f3 channel - shaped pulse 180 degree  (N, selective for Pro)
;p3 : f2 channel -  90 degree high power pulse
;p4 : f2 channel - 180 degree high power pulse
;p6 : f1 channel -  90 degree low power pulse
;p8 : f1 channel - 180 degree shaped pulse for inversion (adiabatic)
;p11: f1 channel -  90 degree shaped pulse
;p12: f1 channel - 180 degree shaped pulse
;p16: homospoil/gradient pulse                  [1 msec]
;p19: gradient pulse 2                          [500 usec]
;p21: f3 channel -  90 degree high power pulse
;p22: f3 channel - 180 degree high power pulse
;p25: f1 channel - 180 degree shaped pulse (Ca, sp28)
;p59: f3 channel - 180 degree shaped pulse (N, selective for Pro)
;d0 : incremented delay (F1 in 3D)              [3 usec]
;d1 : relaxation delay; 1-5 * T1
;d3 : 1/(6J(HCa))                               [950 usec]
;d4 : 1/(4J(HCa))                               [1.8 msec]
;d9 : TOCSY mixing time                         [12 msec]
;d10: incremented delay (F2 in 3D)              [3 usec]
;d11: delay for disk I/O                        [30 msec]
;d12: delay for power switching                 [20 usec]
;d16: delay for homospoil/gradient recovery     [150 usec]
;d22: 1/(4J(COCa))                              [4.5 msec]
;d23: 1/(4J(NCO))                               [16.6 msec]
;cnst21: CO chemical shift (offset, in ppm)
;cnst22: Calpha chemical shift (offset, in ppm)
```

```
;cnst23: Caliphatic chemical shift (offset, in ppm)
;o1p: CO chemical shift (cnst21)
;l0: flag to switch between inphase and antiphase
;l1: loop for FLOPSY16 cycle: ((p6*188.448) * l1) = mixing time
;inf1: 1/SW(Cali) = 2 * DW(Cali)
;inf2: 1/SW(N) = 2 * DW(N)
;in0: 1/(2 * SW(Cali)) =  DW(Cali)
;nd0: 2
;in10: 1/(2 * SW(N)) =  DW(N)
;nd10: 2
;ns: 16 * n
;ds: >= 32
;td1: number of experiments in F1
;td2: number of experiments in F2
;FnMODE: States-TPPI (or TPPI) in F1
;FnMODE: States-TPPI (or TPPI) in F2
;cpd2: decoupling according to sequence defined by cpdprg2
;cpd3: decoupling according to sequence defined by cpdprg3
;pcpd2: f2 channel - 90 degree pulse for decoupling sequence
;pcpd3: f3 channel - 90 degree pulse for decoupling sequence


;for z-only gradients:
;gpz1: 60%
;gpz2: 50%
;gpz3: 19%
;gpz4: 11%

;use gradient files:
;gpnam1: SMSQ10.100
;gpnam2: SMSQ10.100
;gpnam3: SMSQ10.100
;gpnam4: SMSQ10.100


                                        ;preprocessor-flags-start
;LABEL_F2: to do ipap in F2 start experiment with
;             option -DLABEL_F2 (eda: ZGOPTNS)
                                        ;preprocessor-flags-end



;use AU-program splitcomb [ipap 2] (F1 or F2 with LABEL_F2) to process data



;$Id: $
```

Pulse sequence 3 – **3D (H)CBCANCO_pro**

```
;c_hcbcanco_ia3d_pro
;avance-version (20/07/28)
;(H)CbCaNCO
;3D sequence with
;   13C detected correlation for triple resonance using
;      multiple inept transfer steps
;
;      F2(Ha/b) -> F1(Ca/b,t1) -> F1(Ca)
;              -> F3(N,t2) -> F1(C=O,t3)
;
;on/off resonance 13C pulses using shaped pulses
;phase sensitive (t1)
;phase sensitive (t2)
;using constant time in t1
;using constant time in t2
;using IPAP scheme for virtual decoupling
;using selective N pulse for Pro
;(use parameterset )
;
;M.G. Murrali, A. Piai, W. Bermel, I.C. Felli & R. Pierattelli,
;   ChemBioChem 19, 1625-1629 (2018)
;W. Bermel, I. Bertini, V. Csizmok, I. C. Felli, R. Pierattelli &
;   P. Tompa, J. Magn. Reson. 198, 275-281 (2009)
;(W. Bermel, I. Bertini, I.C. Felli, R. Kuemmerle
;   & R. Pierattelli, J. Magn. Reson. 178, 56-64 (2006) )
;
;$CLASS=HighRes
;$DIM=3D
;$TYPE=
;$SUBTYPE=
;$COMMENT=


prosol relations=<triple_c>


#include <Avance.incl>
#include <Grad.incl>
#include <Delay.incl>


"p4=p3*2"
"p22=p21*2"
"d11=30m"
```

```
"d12=20u"
"d3=1.1m"
"d4=1.8m"
"d22=4.5m"
"d23=11.0m"
"d25=4.0m"
"d27=16.0m"
"d0=3u"
"d10=d23"
"d20=d25-p12-4u"
"d30=d27-larger(p12,p59)/2"
"in0=inf1/2"
"in10=inf2/2"
"in20=in0"
"in30=in10"
"td1=tdmax(td1,d20*2,in20)"
"td2=tdmax(td2,d30*2,in30)"
"DELTA1=d25-d0-larger(p12,p22)-d3-p4"
"DELTA2=d27-d10-p12-larger(p12,p59)/2"
"DELTA3=d27/2-p12/2"
"DELTA4=d27-d22-p12"
"l0=1"
"spoff23=0"
"spoff24=0"
"spoff25=0"
"spoff26=bf1*((cnst21-cnst23)/1000000)"
"spoff27=bf1*((cnst22-cnst21)/1000000)"
"o1_F1=bf1*cnst23/1000000"
aqseq 321
1 ze
```

```
 d11 pl12:f2 pl16:f3
2 d11 do:f2 do:f3
3 d12 fq=cnst23(bf ppm):f1
 d1
 d12 pl2:f2 pl3:f3
 50u UNBLKGRAD

 (p3 ph1):f2
 d4
 (center (p12:sp24 ph1) (p4 ph1):f2 )
 d4
 (p3 ph2):f2

 p16:gp1
 d16

 (p11:sp23 ph3)
 d0
 (center (p12:sp26 ph6) (p22 ph1):f3 )
 d3
 (p4 ph1):f2
 DELTA1
 (p12:sp24 ph1)
 d20
 (p12:sp26 ph1)
 4u
 (p11:sp25 ph1)

 d23
 (center (p12:sp24 ph1) (p22 ph1):f3 )
 d23 pl12:f2
 (p11:sp23 ph1)

 p16:gp2
 d16 fq=cnst21(bf ppm):f1
 20u cpd2:f2

 (p21 ph4):f3
 d10
 (p12:sp27 ph6)
 DELTA2
 (center (p12:sp24 ph6) (p59:sp46 ph1):f3 )
 d30 pl3:f3
 (p21 ph1):f3

 4u do:f2
 p16:gp3
 d16
```

```
  20u cpd2:f2

  if "l0 %2 == 1"
    {
    (p11:sp23 ph5)
    DELTA3
    (p12:sp27 ph1)
    DELTA3
    (center (p12:sp24 ph1) (p22 ph1):f3 )
    DELTA3
    (p12:sp27 ph1)
    DELTA3 pl16:f3
    }
  else
    {
    (p11:sp23 ph7)
    d22
    (p12:sp27 ph1)
    DELTA4
    (center (p12:sp24 ph1) (p22 ph1):f3 )
    DELTA3
    DELTA3 pl16:f3
    (p12:sp27 ph1)
    }

  4u BLKGRAD

  go=2 ph31 cpd3:f3
  d11 do:f2 do:f3 mc #0 to 2

#   ifdef LABEL_F1
    F1I(iu0, 2)
#   else
    F2I(iu0, 2)
#   endif /*LABEL_F1*/

    F1PH(calph(ph3, +90), caldel(d0, +in0) & caldel(d20, -in20))
    F2PH(calph(ph4, +90), caldel(d10, +in10) & caldel(d30, -in30))
exit


ph1=0
ph2=1
ph3=0 2
ph4=0 0 2 2
ph5=0 0 0 0 2 2 2 2
ph6=0 0 0 0 0 0 0 0 2 2 2 2 2 2 2 2
ph7=3 3 3 3 1 1 1 1
```

```
ph31=0 2 2 0 2 0 0 2


;pl1 : f1 channel - power level for pulse (default)
;pl2 : f2 channel - power level for pulse (default)
;pl3 : f3 channel - power level for pulse (default)
;pl12: f2 channel - power level for CPD/BB decoupling
;pl16: f3 channel - power level for CPD/BB decoupling
;sp23: f1 channel - shaped pulse  90 degree  (on resonance)
;sp24: f1 channel - shaped pulse 180 degree  (on resonance)
;sp25: f1 channel - shaped pulse  90 degree  (on resonance)
;                 for time reversed pulse
;sp26: f1 channel - shaped pulse 180 degree  (C=O off resonance)
;sp27: f1 channel - shaped pulse 180 degree  (Ca off resonance)
;sp46: f3 channel - shaped pulse 180 degree  (N, selective for Pro)
;p3 : f2 channel -  90 degree high power pulse
;p4 : f2 channel - 180 degree high power pulse
;p11: f1 channel -  90 degree shaped pulse
;p12: f1 channel - 180 degree shaped pulse
;p16: homospoil/gradient pulse                       [1 msec]
;p21: f3 channel -  90 degree high power pulse
;p22: f3 channel - 180 degree high power pulse
;p59: f3 channel - 180 degree shaped pulse (N, selective for Pro)
;d0 : incremented delay (F1 in 3D)                   [3 usec]
;d1 : relaxation delay; 1-5 * T1
;d3 : 1/(6J(HCa))                              [1.1 msec]
;d4 : 1/(4J(HCa))                              [1.8 msec]
;d10: incremented delay (F2 in 3D) = d23
;d11: delay for disk I/O                            [30 msec]
;d12: delay for power switching                      [20 usec]
;d16: delay for homospoil/gradient recovery
;d20: decremented delay (F1 in 3D) = d25-p12-4u
;d22: 1/(4J(COCa))                              [4.5 msec]
;d23: 1/(4J(NCa))                              [11.0 msec]
;d25: 1/(8J'(CaCb))                             [4.0 msec]
;d27: 1/(4J(NCO))                              [16.0 msec]
;d30: decremented delay (F2 in 3D) = d27-larger(p12,p59)/2
;cnst21: CO chemical shift (offset, in ppm)
;cnst22: Calpha chemical shift (offset, in ppm)
;cnst23: Caliphatic chemical shift (offset, in ppm)
;o1p: CO chemical shift (cnst21)
;l0: flag to switch between inphase and antiphase
;inf1: 1/SW(Ca) = 2 * DW(Ca)
;inf2: 1/SW(N) = 2 * DW(N)
;in0: 1/(2 * SW(Ca)) =  DW(Ca)
;nd0: 2
;in10: 1/(2 * SW(N)) =  DW(N)
;nd10: 2
```

```
;in20: = in0
;in30: = in10
;ns: 16 * n
;ds: >= 32
;td1: number of experiments in F1
;td2: number of experiments in F2
;FnMODE: States-TPPI (or TPPI) in F1
;FnMODE: States-TPPI (or TPPI) in F2
;cpd2: decoupling according to sequence defined by cpdprg2
;cpd3: decoupling according to sequence defined by cpdprg3
;pcpd2: f2 channel - 90 degree pulse for decoupling sequence
;pcpd3: f3 channel - 90 degree pulse for decoupling sequence


;for z-only gradients:
;gpz1: 50%
;gpz2: 30%
;gpz3: 19%

;use gradient files:
;gpnam1: SMSQ10.100
;gpnam2: SMSQ10.100
;gpnam3: SMSQ10.100



                                        ;preprocessor-flags-start
;LABEL_F2: to do ipap in F2 start experiment with
;             option -DLABEL_F2 (eda: ZGOPTNS)
                                        ;preprocessor-flags-end



;use AU-program splitcomb [ipap 2] (F1 or F2 with LABEL_F2) to process data



;$Id: $
```

Pulse sequence 4 – **3D (H)CACOCON_pro**

```
;c_hcacocon_ia3d_pro
;avance-version (20/07/28)
;(HCa)COCON-TOCSY
;3D sequence with
;   homonuclear Hartman-Hahn transfer using MOCCA-XY16
;      sequence for mixing
;
;      F2(H) -> F1(Ca) -> F1(C=O,t1, MOCCA) -> F3(N,t2) -> F1(C=O,t3)
;
;on/off resonance 13C pulses using hard and shaped pulses
;phase sensitive (t1)
;phase sensitive (t2)
;using semi-constant time in t1
;using IPAP scheme for virtual decoupling
;using selective N pulse for Pro
;(use parameterset )
;
;M.G. Murrali, A. Piai, W. Bermel, I.C. Felli & R. Pierattelli,
;   ChemBioChem 19, 1625-1629 (2018)
;I.C. Felli, R. Pierattelli, S.J. Glaser & B. Luy,
;   J. Biomol. NMR 43, 187-196 (2009)
;W. Bermel, I. Bertini, I.C. Felli, Y.-M. Lee, C. Luchinat & R.
Pierattelli,
;   J. Am. Chem. Soc. 128, 3918-3919
;S. Balayssac, B. Jimenez & M. Piccioli,
;   J. Magn. Reson. 182, 325-329 (2006)
;
;$CLASS=HighRes
;$DIM=3D
;$TYPE=
;$SUBTYPE=
;$COMMENT=

prosol relations=<triple_c>

#include <Avance.incl>
#include <Delay.incl>
#include <Grad.incl>


"p22=p21*2"
"d11=30m"
"d12=20u"

"d3=1.1m"
"d4=1.8m"
"d22=4.5m"
```

```
"d23=16.6m"

"d21=300u"


"d10=3u"
"d28=3u"
"d0=d22"
"d20=d22+d28-4u"

"in0=inf1/2"
"in10=inf2/2"

"FACTOR1=d20*10000000*2/td1"
"INCR1=FACTOR1/10000000"

"if ( INCR1 > in0 ) { in20 = in0; } else { in20 = INCR1; }"
"if ( INCR1 > in0 ) { in28 = 0; } else { in28=in0-INCR1; }"

"TAU=d21/2"

"FACTOR2=(d9/((d21+p14))*16)"
"l1=FACTOR2*16"

"d31=(d21+p14)*l1"

"DELTA=d10*2+p8"
"DELTA1=d4-larger(p4,p25)/2"
"DELTA2=d22-d3-p4"
"DELTA3=d23/2-p12/2"
"DELTA4=d23-d22-p12"
"DELTA5=d23-larger(p12,p59)/2"

"l0=0"

"spoff13=bf1*((cnst21/2+cnst22/2)/1000000)-o1"
"spoff23=0"
"spoff24=0"
"spoff25=0"
"spoff26=bf1*((cnst22-cnst21)/1000000)"
"spoff27=bf1*((cnst21-cnst22)/1000000)"


"o1_F1=bf1*cnst21/1000000"


aqseq 321


1 ze
  d11 pl12:f2 pl16:f3
2 d11 do:f2 do:f3
```

```
  4u BLKGRAD
  d1 pl1:f1 pl2:f2
  20u rpp11
  50u UNBLKGRAD
  d12 cpd3:f3
  20u fq=cnst22(bf ppm):f1

  (p3 ph1):f2
  DELTA1
  (center (p25:sp28 ph1) (p4 ph1):f2 )
  DELTA1
  (p3 ph2):f2

  (p11:sp23 ph1)
  d3
  (p4 ph1):f2
  DELTA2
  (p12:sp27 ph1)
  4u
  (p25:sp28 ph1)
  d22
  (p12:sp27 ph1)
  4u
  (p11:sp25 ph5)

  p16:gp4
  d16 fq=cnst21(bf ppm):f1

  (p11:sp23 ph3)
  d0
  (p12:sp26 ph1)
  d28
  (p12:sp24 ph1)
  d20
  (p12:sp26 ph1)
  4u
  (p11:sp25 ph4)

  4u do:f3
  p16:gp1
  d16 pl20:f1

                                ;begin MOCCA-XY16
4 TAU
  (p14 ph11)
  TAU ipp11
  lo to 4 times l1
                                ;end MOCCA-XY16

  p16:gp2
  d16 pl12:f2 pl3:f3
  20u cpd2:f2
```

```

   (p11:sp23 ph1)
   DELTA5
   (center (p12:sp24 ph1) (p59:sp46 ph1):f3 )
   DELTA5
   (p11:sp25 ph2)

   4u do:f2
   p16:gp3
   d16 pl3:f3
   20u cpd2:f2

   (p21 ph6):f3
   d10
   (p8:sp13 ph1)
   d10
   (p22 ph1):f3
   DELTA
   (p21 ph1):f3

   if "l0 %2 == 0"
      {
      (p11:sp23 ph1)
      DELTA3
      (p12:sp26 ph1)
      DELTA3
      (center (p12:sp24 ph1) (p22 ph1):f3 )
      DELTA3
      (p12:sp26 ph1)
      DELTA3 pl16:f3
      }
   else
      {
      (p11:sp23 ph7)
      d22
      (p12:sp26 ph1)
      DELTA4
      (center (p12:sp24 ph1) (p22 ph1):f3 )
      DELTA3
      DELTA3 pl16:f3
      (p12:sp26 ph1)
      }

   4u BLKGRAD

   go=2 ph31 cpd3:f3

   d11 do:f2 do:f3 mc #0 to 2

#   ifdef LABEL_F1
      F1I(iu0, 2)
#   else
```

```
     F2I(iu0, 2)
#   endif /*LABEL_F1*/

     F1PH(calph(ph3, +90), caldel(d0, +in0) & caldel(d20, -in20) &
caldel(d28, +in28))
     F2PH(calph(ph6, +90), caldel(d10, +in10))

  d31
exit


ph1=0
ph2=1
ph3=0 2
ph4=1 1 1 1 1 1 1 1 3 3 3 3 3 3 3 3
ph5=0 0 0 0 2 2 2 2
ph6=0 0 2 2
ph7=3
ph11=0 1 0 1 1 0 1 0 2 3 2 3 3 2 3 2
ph31=0 2 2 0 2 0 0 2 2 0 0 2 0 2 2 0


;pl1 : f1 channel - power level for pulse (default)
;pl2 : f2 channel - power level for pulse (default)
;pl3 : f3 channel - power level for pulse (default)
;pl12: f2 channel - power level for CPD/BB decoupling
;pl16: f3 channel - power level for CPD/BB decoupling
;pl20: f1 channel - power level for TOCSY (high sel.)
;sp13: f1 channel - shaped pulse 180 degree  (adiabatic)
;sp23: f1 channel - shaped pulse  90 degree  (on resonance)
;sp24: f1 channel - shaped pulse 180 degree  (on resonance)
;sp25: f1 channel - shaped pulse  90 degree  (on resonance)
;                   for time reversed pulse
;sp26: f1 channel - shaped pulse 180 degree  (Ca off resonance)
;sp27: f1 channel - shaped pulse 180 degree  (C=O off resonance)
;sp28: f1 channel - shaped pulse 180 degree  (Ca on resonance)
;                   sp28 requires higher selectivity than sp24
;sp46: f3 channel - shaped pulse 180 degree  (N, selective for Pro)
;p3 : f2 channel -  90 degree high power pulse
;p4 : f2 channel - 180 degree high power pulse
;p8 : f2 channel - 180 degree shaped pulse for inversion (adiabatic)
;p11: f1 channel -  90 degree shaped pulse
;p12: f1 channel - 180 degree shaped pulse
;p14: f1 channel - 180 degree low power pulse at pl20
;p16: homospoil/gradient pulse                         [1 msec]
;p21: f3 channel -  90 degree high power pulse
;p22: f3 channel - 180 degree high power pulse
;p25: f1 channel - 180 degree shaped pulse (Ca, sp28)
;p59: f3 channel - 180 degree shaped pulse (N, selective for Pro)
;d0 : incremented delay (F1 in 3D): = d22
;d1 : relaxation delay; 1-5 * T1
;d3 : 1/(6J(HCa))                                      [1.1 msec]
```

```
;d4 : 1/(4J(HCa))                                    [1.8 msec]
;d9 : TOCSY mixing time
;d10: incremented delay (F2 in 3D)                   [3 usec]
;d11: delay for disk I/O                             [30 msec]
;d12: delay for power switching                      [20 usec]
;d16: delay for homospoil/gradient recovery
;d20: decremented delay (F1 in 3D): = d22+d28-4u
;d21: delay for MOCCA sequence                       [300 usec]
;d22: 1/(4J(COCa))                                   [4.5 msec]
;d23: 1/(4J(NCO))                                    [15.0 msec]
;d28: incremented delay (F1 in 3D)                   [3 usec]
;d31: total mixing time as executed
;cnst21: CO chemical shift (offset, in ppm)
;cnst22: Calpha chemical shift (offset, in ppm)
;o1p: CO chemical shift (cnst21)
;l0: flag to switch between inphase and antiphase
;l1: loop for MOCCA-XY16 cycle: ((TAU*2+p14)*16 * l1) = mixing time
;inf1: 1/SW(CO) = 2 * DW(CO)
;inf2: 1/SW(N) = 2 * DW(N)
;in0: 1/(2 * SW(CO)) =  DW(CO)
;nd0: 2
;in10: 1/(2 * SW(N)) =  DW(N)
;nd10: 2
;in20: = k * in0
;in28: = (1 - k) * in0
;ns: 8 * n
;ds: >= 32
;td1: number of experiments in F1
;td2: number of experiments in F2
;FnMODE: States-TPPI (or TPPI) in F1
;FnMODE: States-TPPI (or TPPI) in F2
;cpd2: decoupling according to sequence defined by cpdprg2
;cpd3: decoupling according to sequence defined by cpdprg3
;pcpd2: f2 channel - 90 degree pulse for decoupling sequence
;pcpd3: f3 channel - 90 degree pulse for decoupling sequence

;for z-only gradients:
;gpz1: 80%
;gpz2: 70%
;gpz3: 30%
;gpz4: 50%

;use gradient files:
;gpnam1: SMSQ10.100
;gpnam2: SMSQ10.100
;gpnam3: SMSQ10.100
;gpnam4: SMSQ10.100

;use AU-program splitcomb [ipap 2] to process data

;$Id: $
```
